# Assessing the Variability of Heavy Metal Concentrations in Liquid-Solid Two-Phase and Related Environmental Risks in the Weihe River of Shaanxi Province, China

**DOI:** 10.3390/ijerph120708243

**Published:** 2015-07-17

**Authors:** Jinxi Song, Xiaogang Yang, Junlong Zhang, Yongqing Long, Yan Zhang, Taifan Zhang

**Affiliations:** 1 State Key Laboratory of Soil Erosion and Dryland Farming on the Loess Plateau, Institute of Soil and Water Conservation, Chinese Academy of Sciences, Yangling 712100, China; 2 College of Urban and Environmental Sciences, Northwest University, Xi’an 710027, China; E-Mails: yxgyff166@163.com (X.Y.); junlongzhangcq@hotmail.com (J.Z.); sjzxlyq@nwu.edu.cn (Y.L.); zhangy.11b@igsnrr.ac.cn (Y.Z.); zhangtaifan@163.com (T.Z.)

**Keywords:** heavy metal, hyporheic sediment, seasonal, liquid-solid two-phase, benthos activity, risk

## Abstract

Accurate estimation of the variability of heavy metals in river water and the hyporheic zone is crucial for pollution control and environmental management. The biotoxicities and potential ecological risks of heavy metals (Cu, Zn, Pb, Cd) in a solid-liquid two-phase system were estimated using the Geo-accumulation Index, Potential Ecological Risk Assessment and Quality Standard Index methods in the Weihe River of Shaanxi Province, China. Water and sediment samples were collected from five study sites during spring, summer and winter, 2013. The dominant species in the streambed sediments were chironomids and flutter earthworm, whose bioturbation mainly ranged from 0 to 20 cm. The concentrations of heavy metals in surface water and pore water varied obviously in spring and summer. The degrees of concentration of Cu and Cd in spring and summer were higher than the U.S. water quality Criteria Maximum Concentrations. Furthermore, the biotoxicities of Pb and Zn demonstrated season-spatial variations. The concentrations of Cu, Zn, Pb and Cd in spring and winter were significantly higher than those in summer, and the pollution levels also varied obviously in different layers of the sediments. Moreover, the pollution level of Cd was the most serious, as estimated by all three assessment methods.

## 1. Introduction

The hyporheic zone is the saturated area between surface water and groundwater [1]. As a transitional belt between the surface water and groundwater systems [2], it plays an essential role in the water exchange process, hydrologic regulation, environmental buffer and ecological protection [3]. The study on the hyporheic zone started from biological research and measurement, and its study gradually developed from a static research stage into a dynamic one [4]. Since then, the ecology of hyporheic zone has been one of important research areas in the hydrological water exchange process. In China, many researchers originally focused on the study of the hydrological roles of hyporheic zone rather than the biological significance of this zone. Since the Industrial Revolution, river ecology has experienced a period of rapid development with the deterioration of river pollution [5]. Heavy metals have become essential elements disturbing the normal functions of a stream due to their characteristics of serious toxicity, difficult degradation and easy concentration [6]. This usually causes secondary pollution if heavy metals in sediments are discharged through a series of biological, physical and chemical processes when the sedimentary conditions are changed [7]. Therefore, the heavy metal concentrations in sediments have been regarded as one of the important indices to determine the aquatic quality of a river [8], which has become one of major concerns in water environment research [9]. Many studies on heavy metals in rivers have been conducted and many great results have been published in the literature, such as spatial distributions of heavy metals in streambeds, sources and ecological assessment [10,11,12,13]. However, these studies focused on the horizontal distribution at a surface depth between 0 cm and 10 cm and the vertical activity range of aquatic benthos was not considered. Also, there is lack of information on the temporal scale of the integrated toxicity of heavy metals in liquid-solid two phase systems is scarce.

Situated in the central area of the “Guanzhong-Tianshui” economic zone in China, the Weihe River basin occupies an important position in the social and economic development outlined in China’s Western Development Strategy [14]. Recently, due to the increase of population, agriculture and industries in the area, the conflict between human development and aquatic environment has been serious, limiting the sustainability of the riverine ecology [15]. There are many studies involving the heavy metal levels in the Weihe River. The instream flow requirements are of great importance in maintaining the river health and ecosystem. It is conductive for river ecological restoration [16]. The sediment load of the Weihe River decreases stepwise in the reaches of the Yellow River, and this is influenced by the climate change and human activities [17,18]. Additionally, the water discharge has clear seasonal attributes [19]. These are the factors related with the concentration and transport heavy metals of the river. The heavy metal pollution is increasing in some terms due to the intensive human disturbance in the Wei River basin [15,20], and it has become a seasonal river with an unpleasant status, but the research on heavy metal contaminants in this river is relative weak [16]. The hyporheic zone provides a refuge for invertebrates [21], and variability of heavy metals in this zone could alter the invertebrates’ community structure of the river because the biota indictors can reflect the water quality [22,23]. However, these studies have their limitations in the following aspects: (1) Only the sediments in the surface depth (0–10 cm) were investigated, which did not consider the vertical variation features of heavy metals and the active areas of the benthos; (2) Evaluation results couldn’t reflect the whole status and seasonal change of heavy metals in sediments of the river due to insufficient sampling spots and seasons; (3) The hydraulic exchange features in hyporheic zone and the relations of heavy metals in the liquid-solid two-phase system were not included in the studies. Therefore, it is of great importance in assessing the variability of heavy metal concentrations in the liquid-solid two-phase system and related environmental risks for the management of water resources.

Accordingly, the present study assesses the concentrations variability of four heavy metals in the liquid-solid two-phase region and related environmental risks through investigation of the sediments and pore water in the Weihe River of Shaanxi Province. The aims of this study were: (1) determining the activity ranges of benthos under the heavy metal pollution; (2) illustrating the spatial and seasonal variations of the heavy metals in spring, summer and winter; and (3) evaluating the related environmental risks.

## 2. Materials and Methods

### 2.1. Study Area Description

The Weihe River, the largest tributary of the Yellow River, originates from the Niaoshu Mountain in Weiyuan county of Gansu Province, China. With a total length of 818 km, the river runs across Shaanxi, Gansu and Ningxia provinces, and finally flows into the Yellow River in Tongguan of Shaanxi Province. The Weihe River basin covers an area of 13.5 × 10^4^ km^2^, of which an area of 6.67 × 10^4^ km^2^ is in Shaanxi Province, taking account of 49.4% of the whole basin area. Average annual flow of the river in the province is 103.7 × 10^8^ m^3^, average annual sediment yield 5.8 × 10^8^ t, accounting for 1/3 of the Yellow River sediment transport. The river basin covers three topographic classes, namely Loess Plateau, the Guanzhong Plain and Qinling Mountains. The basin belongs to a typical continental monsoon climate with cold winters and dry, hot and rainy summers, where heavy rains mainly happen from June to October. In addition, the majority of the river basin is covered with loose loess, which has caused serious sediment transportation and vulnerable erosion areas [16].

### 2.2. Sampling Methods 

**Figure 1:**
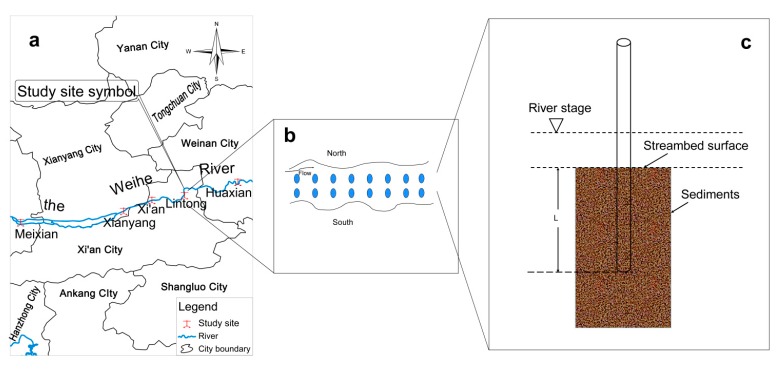
(**a**) The study area, (**b**) sample spots and (**c**) sampling method in the Weihe River.

Five study sites in the Weihe River of Shaanxi Province was chosen for sample collection, namely Meixian (34°14′31.13″ N, 107°48′43.63″ E), Xianyang (34°18′33.48″ N, 108°40′36.96″ E), Xi’an (34°22′28.63″ N, 108°50′01.70″ E), Lintong (34°25′13.52″ N, 109°10′43.53″ E), and Huaxian (34°34′44.46″ N, 109°40′35.14″ E) (Figure 1a). Due to the continuous rainfall that limited the field work in autumn, the sediment samples were collected from these five sites in spring, summer and winter of 2013. Arrangement of the sampling points in each gauge are influenced significantly by different topography and hydrological conditions (Figure 1b), and the water depth of river channel in the gauge, thus the distances of sampling points to riverbank should be unequal. We arranged the sampling spots randomly along the river flowing direction using PVC pipes with unequal distances to the riverbank ranging from 1 m to 5 m (Figure 1c). The PVC pipes were arranged in two rows, where one row was used to study the vertical activity ranges of the benthos and another to analyze the concentration of the heavy metals in liquid-solid phase. In order to facilitate the comparative study, the PVC pipes in two rows were paired and the distances between the paired pipes were controlled within 50 cm. The detailed process of sampling using PVC pipes was described as follows. Firstly, a transparent PVC pipe (1.6 m in length, 54 mm in inner diameter, 60 mm in outside diameter) was inserted vertically into the streambed in the depth about 40 cm. Secondly, the pipes were filled with river water from the upper end to prevent the sediments from being scattered, and then the top end of the pipe was sealed using a rubber stopper. Thirdly, the pipes were pulled out vertically from the riverbed sediments. Finally, the PVC pipes for activity ranges of the benthos were numbered and brought back to the laboratory for analysis. The PVC pipes for the concentrations of the heavy metals were grouped and numbered according to the samples from different layers, including 0–10 cm, 10–20 cm, 20–30 cm, and 30–40 cm, and then they were sent back to the laboratory for test with polyethylene closure bags.

### 2.3. Sampling Analysis

In the laboratory, each sample in the PVC pipe for the activity ranges of the benthos were divided into different layers and the layers classification was similar to the layers classification for the heavy metal concentration samples, and thus the benthos in different layers were determined. The testing range of heavy metals in the liquid-solid two-phase was determined based on the activity ranges of benthos, especially the heavy metals in the layers which were disturbed by benthos and their activities. We abstracted the pore water from the layered samples with high-speed centrifugation method (5000 r·min^−1^ in 30 min). The samples of sediments remaining after centrifugation were pulled out into paper plates to dry naturally. The dried sedimentary matters were mixed equally and re-sampled from two diagonally opposite quarters using a quartering method, from which the test samples of sediments were obtained through 100 mesh sieve. The methods used to analyze the concentrations of heavy metals (Cu, Zn, Pb and Cd) in sediments included HNO_3_-HF (5 mL HNO_3_, 3 mL HF) microwave digestion, electrothermal plate exposure to remove acid, and adding 2% HNO_3_ to a constant volume to 100 mL. For the measurement of the Zn lanthanum nitrate solution was added in order to reduce the influence derived from excessive amounts of Fe. To reduce the non-characteristic absorption due to high salt levels, the background value was neglected to obtain the available data resource. The concentrations of heavy metals (Cu, Zn, Pb and Cd) were determined using an Inductively Coupled Plasma-Optical Emission Spectrometer (ICP-OES, PerkinElmer Co. Ltd., Massachusetts, MA, USA). Using quality control standards calibration curves were produced which were used to evaluate sampling data [11]. Reagents, procedural blanks, and samples were measured six times in parallel and the average of the last three values were used to calculate the results, because the first three were used to clean the pipe to avoid pollution caused by the last sample [24]. The potassium dichromate volumetric method was used to test the content of organic matter (OM), determination of total nitrogen (TN) was performed by the alkaline potassium persulfate digestion-UV spectrophotometric method, and total phosphorus (TP) was measured by molybdate spectrophotometry.

### 2.4. Evaluation Methods for Heavy Metals

#### 2.4.1. Geo-Accumulation Index (GAI)

The geo-accumulation index was developed by Muller based on his study on heavy metals in fluvial stream sediments [25]. It has been widely used because it takes into account the effects of the natural elements and human activities on pollution [26]. The geo-accumulation index is expressed by following Equation:

(1) $$I_{geo}=\log_{2}\left[C_{i}/1.5B_{i}\right]$$

where I_geo_ is the geo-accumulation index, $C_{i}$ is the measured concentration of a heavy metal *i* in sedimentary matter, $B_{i}$ is the geochemical background value of the heavy metal *i*.

The assessment results are greatly influenced by the reference values [10]. Fully considering the discrepancies of heavy metals’ background values in the sediments of the Weihe River, we chose the background values in different gauges based on the weight value before industrialization in Shaanxi Province (*i.e.*, contents of heavy metal in the sediments were expressed by quality fraction (W) (mg·kg^−1^)) (Table 1), which can guarantee the evaluation results to reflect the real stream pollution situation [27]. 

**Table 1:** The background values and toxic response coefficients of the heavy metals in streambed sediments of the Weihe River in Shaanxi Province.

Heavy Metals	Background Value (mg·kg^−1^)				Toxicity Coefficient of Heavy Metals
	Shaanxi Province	Baoji	Xianyang	Xi’an	
W(Cu)	21.4	22.6	24.0	20.1	5
W(Zn)	69.4	74.2	64.4	66.1	1
W(Pb)	21.4	26.0	16.9	20.9	5
W(Cd)	0.094	0.097	/	/	30

In comparison to [25], we classified the pollution levels of the heavy metals in sediments into seven levels based on the geo-accumulation index ranges (Table 2).

**Table 2:** The values of geo-accumulation index and its related contamination levels for heavy metals (mg·kg**^−^**^1^).

I_geo_ Index	I_geo_ Class	Pollution Level	I_geo_	I_geo_ Class	Pollution Level
<0	0	No	3–4	4	Serious
0–1	1	Low	4–5	5	Very serious
1–2	2	Moderate	>5	6	Extremely serious
2–3	3	Less serious			

#### 2.4.2. Potential Ecological Risk Assessment (PERA)

Potential ecological risk assessment is an index method used to quantitatively assess the potential ecological hazards of heavy metals in the sediments according to the features of the heavy metals and their environmental behavior [10]. Due to its advantages of considering the heavy metal toxicity, reflecting the comprehensive impacts of different pollutants, and distinguishing quantitatively the level of potential ecological hazards [28], this method have been widely employed to evaluate the ecological impacts of the heavy metals in the sediments in rivers, lakes and reservoirs, intertidal zones [29]. It can be expressed by Equation (2):

(2) $$E_{RI}=\overset{m}{\underset{i}{\sum }}E_{r}^{i}=\overset{m}{\underset{i}{\sum }}T_{r}^{i}\cdot C_{f}^{i}=\overset{m}{\underset{i}{\sum }}T_{r}^{i}\cdot C^{i}/C_{n}^{i}$$

where$\;E_{RI}\;$is the comprehensive index of potential ecological risk of multiple heavy metals; $C^{i}\;$is the measured value of the heavy metal *i*; $C_{n}^{i}\;$is the reference value; $C_{f}^{i}\;$is the pollution coefficient of the heavy metal *i*; $T_{r\;}^{i}$is the toxicity coefficient of the heavy metal *i*, which reflects the toxicity level of heavy metal *i* and the sensitivity degrees of aquatic organisms to heavy metal pollution (Table 1); $E_{r}^{i}\;$is the coefficients of the potential ecological hazard of heavy metal *i*.

The highest background value of a heavy metal in sediments before the industrialization stage is usually applied as the reference value. However, background values of heavy metals usually differ in different study areas, therefore selecting different background values will significantly influence the results of the potential ecological risk assessment. In this study, we chose the most suitable background values as the reference values, which were able to accurately reflect the real situations of heavy metals in the Weihe River in Shaanxi Province (Table 1). The estimation and hazard levels classification of $E_{RI}$ are usually based on eight main heavy metals, including polychlorinated biphenyls (PCBs), Hg, Cd, As, Pb, Cu, Cr and Zn [30,31]. Due to the limitations in the laboratory and real stream situations, researchers usually test only certain parameters, thus the parameters of hazard levels classification should be revised in order to obtain an objective and real estimation result [31,32]. This study evaluated four main heavy metals for the Weihe River, including Cu, Zn, Pb and Cd, and we adjusted the classification standards of evaluation indexes [10] including *E_i_*, *E_RI_* and the corresponding levels of ecological hazards according to types and amounts of heavy metals (Table 3).

**Table 3:** The levels of potential ecological hazard of heavy metals (mg·kg^−1^).

Ei	RI	Pollution Intense
$E_{r}^{i\;}$ < 30	E_RI_ < 50	Unpolluted
30 ≤ $E_{r}^{i}$ < 60	50 ≤ E_RI_ < 100	Moderate
60 ≤ $E_{r}^{i}$ <120	100 ≤ E_RI_ < 200	Strong
120 ≤ $E_{r}^{i}$ < 240	E_RI_ ≥ 200	Very strong
$E_{r}^{i}$ ≥ 240	–	Extremely strong

#### 2.4.3. Quality Standard Index (QSI)

Sediment quality standard assessment indexes or values were developed using a weight of evidence approach, in which matching biological and chemical data on freshwater sediments from numerous modelling, laboratory, and field studies were compiled and analyzed. To overcome the limitations that chemical analysis methods are unable to judge the specific toxicity of a heavy metal for a certain organism, we combined the data of concentrations and toxicity of heavy metals in sediments for the environmental hazard assessment. The database of the ecological effects (DEE) established on the biological effects of contaminants has continuously improved sediment quality criteria [33]. Meanwhile, toxicity experimental analyses (TEA) has also improved sediment quality criteria [34]. The common characters of the DEE and TEA are based on a large number of chemical data or corresponding biological data, and their purposes are to evaluate the potential toxicity of heavy metals in contaminated sediments. The sedimentary quality criteria have recently been widely used in the research, and national and regional water resource protection and management [35]. In this study, we used the Threshold Effect Level (TEL) and Probable Effect Level (PEL) in the Canadian Quality Criteria of heavy metals in freshwater sediments to evaluate contamination level of heavy metals in the sediments of the Weihe River in Shaanxi Province (Table 4), because freshwater sediment quality standards are currently in a preliminary development stage subject to many arguments in China, and the Canadian quality criteria is commonly used by most researchers and it is proved that it is suitable for China. It indicates that a sediment has been severely contaminated with serious toxicity to organisms when the concentration of a heavy metal in the sediments is greater than the PEL. In contrast, it suggests that the sediment has been lightly polluted or unpolluted when the concentration of the heavy metal is lower than the TEL, which has light or no biological toxicity effects. It shows that the sediment has a moderate pollution when the concentration lies between TEL and PEL, and there is an equal probability of this heavy metal producing toxicity and non-toxicity. Criteria Continuous Concentration (CCC) and Criteria Maximum Concentration (CMC) aim to estimate the impact of the long-term and short-term pollution on aquatic organisms [36]. They were used to assess the heavy metals in surface and pore water. 

**Table 4:** Canadian freshwater sediment guidelines for heavy metals (mg·kg^−1^).

Item	Cu	Zn	Pb	Cd
TEL	36	123	35	0.6
PEL	197	315	91	3.5

## 3. Results and Discussion 

### 3.1. The Investigation of the Activity Ranges of Benthos under the Heavy Metal Pollution 

Aquatic insects account for 78.4% of the total 116 macrobenthos species in the Weihe River in Shaanxi Province. The main benthos included chirognomy, caddis, flutter earthworm, and snails. The observed result on the biological moving traces in PVC pipes found that there were discrepancies between the disturbance depths of different benthos, mainly ranging from 4 to 26 cm (Table 5). We identified the benthos samples collected from five gauges of the Weihe River in Shaanxi Province in spring and summer, and the results showed that the chironomid larvae and tubificidae accounted for 94.7%–100%, except for the Huaxian gauge where no benthos were found, the main benthos in the bottom of the Weihe River basin were annelids, mollusks, arthropods and aquatic insects, among which the majority were chironomidae and tubificidae. Our study results matched those of a previous study [22]. Through the analysis of the vertical activity range of the benthos of different gauges of the Weihe River in Shaanxi Province in spring and summer, we found that the main activity range was distributed at the depths of 0–10 cm and 10–20 cm and even at a depth of 20–30 cm in Meixian gauge in summer. Except for Huaxian gauge, the numbers of benthos decreased with depth. Previous studies revealed that the disturbance of the benthos activities would cause micro-topography changes in the streambed [37], and also impacted the sediment permeability and easily caused substance mixing at different depths [5,38]. Disturbance of the benthos activities were also able to cause the mixtures of heavy metals, nutrients and organic pollutants to transfer downward into deeper layers in the sediments, which expanded their impacts in depth and breadth [39]. This study revealed that the concentration of a single heavy metal displayed a significantly positive correlation in the vertical direction in the hyporheic zone (Table 6), and this was probably because of the sediment exchanges and benthos disturbances in this zone.

**Table 5:** Changes of benthic fauna with streambed depth in the Weihe River of Shaanxi Province.

Gauge Season		Vertical Sediment Biomass					
		0–10 cm		10–20 cm		20–0 cm	
		Range	Mean	Range	Mean	Range	Mean
Meixian	Spring	1–13	Null	0–5	1.7	0	0
	Summer	1–40	10.4	0–3	1	0–2	0.7
Xianyang	Spring	5–58	34.7	0–4	1.4	0	0
Xi’an	Spring	11–95	34.6	0	0	0	0
	Summer	0–20	7.3	0–3	0.7	0	0
Lintong	Spring	0–27	8	0–3	0.4	0	0
	Summer	53–210	107	0–22	4.4	0	0
Huaxian	Spring	0	0	0	0	0	0
	Summer	0	0	0	0	0	0

**Table 6:** Correlation matrix of a single heavy metal in streambed sediments vertically.

W(Cu)	10 cm	120 cm	20-30 cm	W(Zn)	0–10 cm	120 cm	230 cm
0–10 cm	1			0–10 cm	1		
10–20 cm	0.693 **	1		10–20 cm	0.631 **	1	
20–30 cm	0.712 **	0.719 **	1	20–30 cm	0.602 **	0.604 **	1
W(Pb)	10 cm	10–20 cm	20–30 cm	W(Cd)	0–10 cm	10–20 cm	20–30 cm
0–10 cm	1			0–10 cm	1		
10–20 cm	0.981 **	1		10–20 cm	0.805 **	1	
20–30 cm	0.936 **	0.913 **	1	20–30 cm	0.843 **	0.773 **	1

*N* = 73, ** *p* < 0.01 (Two-sided test).

### 3.2. The Spatial and Seasonal Variations of the Heavy Metals

#### 3.2.1. Surface Water

The content of dissolved heavy metals in surface water ranged between 4.1 and 2901 μg·L^−1^ in spring, 1.90–2222 μg·L^−1^ in summer and 27.8–306.1 μg·L^−1^ in winter, respectively. Compared to the National Surface Water Standard of China (GB3838-2002) [40], according to the protection aim and the environmental function of the surface water, the surface water is divided into five levels by this standard, there are the specific values for Cu, Zn, Pb and Cd in each level (Table 7). The mean contents of Pb and Cd were worse than the Grade V standard, indicating that the water quality was poor in the Weihe River. Except for Pb, the concentrations of other metals (Cu, Zn and Cd) in surface water in spring and summer displayed a similar trend along the river flow direction: decreasing at the beginning and then increasing in the Meixian and Lingtong gauges, and declining again. The increasing variation in the Meixian and Lingtong gauges was mainly due to Cu, Zn and Cd loadings in these gauges, and the decrease after these two gauges was mainly due to insignificant pollution sources along the way, the dilution of the water and adsorption of sediment particles. This research also found that the contents of the heavy metals were significantly greater in spring than in summer, except for a few experimental spots. This is may be due to a sudden rainfall followed by high discharge from the upstream environment of the river [41,42]. Comparing the concentrations of Cu, Zn, Pb and Cd in the study area with the concentrations of these four heavy metals in the CCC and the U.S. water quality CMC [36], the results indicated that the Cu concentrations were higher than the CCC and CMC in spring, summer and winter, Zn concentrations were greater than CMC in spring but lower than the CCC level in summer, except for the Lintong gauge. Pb concentrations were greater than the CMC except for Lintong in spring; while the concentrations in summer were lower than the CCC in Meixian and lower than the CMC in Xi’an, but greatly higher than the CMC in Lintong and Huaxian. For Cd, the concentrations were all greater than the CMC in four gauges in spring, summer and winter (Table 7). There will be chronic toxicity to the benthos when the concentration of heavy metals is greater than the CCC, and acute toxic effects will be produced on river aquatic organisms when the concentration level is greater than the CMC, which results in potential hazards to the surrounding soils [43]. Therefore, in comparison of Zn and Pb in the surface water, the hazard levels of Cu and Cd are more serious.

**Table 7:** Contents of heavy metals in the surface water and pore water of the Weihe River in Shaanxi Province (μg·L^−1^).

Water	Gauge	Season	Cu	Zn	Pb	Cd
Surface water	Meixian	Spring	228.3	631.0	135.1	95.7
		Summer	165.7	47.9	1.9	408.4
		Winter	265.3	65	56.3	33.4
	Xianyang	Spring	115.2	477.4	360.4	39.2
		Winter	224.5	81.4	70.4	27.8
	Xi’an	Spring	1168.6	2901.0	1909.1	81.0
		Summer	14.6	14.3	46.2	4.3
		Winter	163.3	106.3	70.4	50.1
	Lintong	Spring	253.3	686.3	43.0	6.2
		Summer	135.0	535.0	2222.0	131.0
		Winter	306.1	69.1	70.4	89.2
	Huaxian	Spring	91.3	291.1	153.2	4.1
		Summer	13.8	9.8	571.4	24.1
		Winter	265.3	60.9	70.4	89.2
Pore water	Meixian	Summer	107.5	24.04	1.48	362.13
		Winter	1108.2	606.5	484.8	463.9
	Xi’an	Summer	19.08	56.29	86.68	5.73
		Winter	530.4	332.2	816.8	366
	Huaxian	Summer	173.67	176.33	941.22	388.11
		Winter	728.9	382.4	672.8	463.8
	Lintong	Summer	8.14	7.98	623.59	18.06
		Winter	1676.5	680.4	575.7	247.7
GB3838-2002(I/II/III/IV/V)			10/1000/1000/1000/1000	50/1000/1000/2000/2000	10/10/50/50/100	1/5/5/5/10
CCC			9	120	2.5	0.25
CMC			13	120	65	2

#### 3.2.2. Pore Water

The pore water is the soluble water contained in pore spaces between the grains of rock and sediments [44]. The heavy metals in pore water were a direct result of the organic morphology in the sediments in the hyporheic zone, whose contents are able to directly indicate the chemical reactivity of heavy metals. The contents of bioavailable heavy metals in systems of water surface sediments determine the potential hazard levels of heavy metals [45,46], and the sedimentary toxicity showed a good positive correlation with the reactivity of the toxic heavy metals in the pore water [7]. The heavy metal contents in pore water ranged from 1.48 to 941.22 ug·L^−1^ during summer in the Weihe River. Compared to the U.S. national recommended water quality criteria of priority pollutants [36], the average concentrations of Cu, Pb and Cd were much higher than the CMC, at 5.93 times, 6.36 times and 96.76 times of the maximum concentrations in the standard, respectively. The contents of heavy metals were quite different in different gauges. Cu contents were all greater than that the CMC in all gauges except the Cu content in the Huaxian gauge was lower than that in the CCC, whereas the Cu content in Lintong was 13.36 times the CMC, 8.27 times in Huaxian, and 1.47 times in the Xi’an gauge. As for Zn, the contents were less than the CCC in gauges except that the content in Lintong gauge, which was greater than the CMC. With reference to Pb, the contents were higher than the CMC in all gauges except the Meixian gauge where it was less than the CCC. The contents were 14.48 times, 5.59 times and 1.33 times of CMC value in the Lintong gauge, Huaxian and Xi’an gauges, respectively. Cd contents were greater than the CMC in all gauges, namely 186.07 times, 2.87 times, 194.06 times and 9.03 times the CMC in the Meixian, Xi’an, Lintong and Huaxian gauge, respectively. These results confirmed that there were also obvious differences between the heavy metal contents in the sedimentary pore water in the study area. Meanwhile, the comparison of the heavy metals of pore water for all the testing sites in summer and winter, showed that the concentrations of heavy metals in winter were greater than in summer and further higher than the CMC for the vast majority of sites, with significant seasonal variations. The correlation analysis showed that correlations between contents of a single heavy metal existed in surface water, pore water and shallow groundwater, and there was mutual conversion and migration (Table 8). Because a large numbers of benthos, especially tubificidae and chironomids were living in the sedimentary matter, heavy metals with high contents like Cu, Pb and Cd in pore water could be accumulated in organisms through bioaccumulation, which harmed the benthos health [47]. Meanwhile the benthos, especially earthworms, earthworm chatter, chironomids, were important bait of fish, and bioaccumulation of these heavy metals also affected the health of fish, which finally influences human health through food chain accumulation as the enrichment range of the heavy metals would expand through the activities of the fish [11], and then influence human health [48]. 

**Table 8:** Correlation matrix of heavy metals in surface water, pore water and groundwater of the Weihe River in Shaanxi Province.

Metals	Water		
	Surface Water (SW)	Pore Water (PW)	Groundwater (GW)
**Cu/SW**	1	0.87 *	0.99 *
**Cu/PW**		1	0.88 *
**Cu/GW**			1
**Zn/SW**	1	0.96 *	0.93 *
**Zn/PW**		1	0.99 **
**Zn/GW**			1
**Pb/SW**	1	0.92 *	0.99 **
**Pb/PW**		1	0.96 *
**Pb/GW**			1
**Cd/SW**	1	0.76 *	0.97 *
**Cd/PW**		1	0.59 *
**Cd/GW**			1

*N* = 12, * *p* < 0.05, ** *p* < 0.01 (Two-sided test).

#### 3.2.3. Seasonal Features of Heavy Metal Concentration Changes 

**Figure 2:**
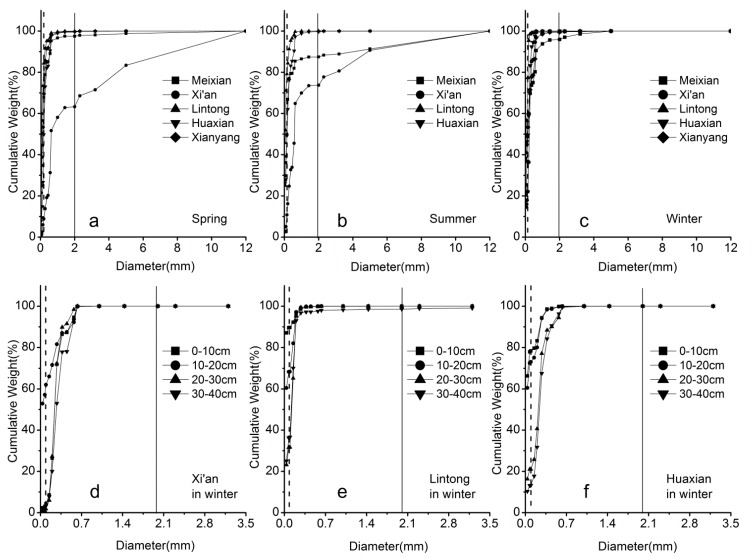
The distribution of grain size for the sediments of the Weihe River (The line indicates specific diameter: d = 0.05 mm, d = 2 mm).

The heavy metal concentrations display greater seasonal differences in the sampling sites and the variation range is relatively greater. In general, the concentrations of heavy metals in spring and winter are greater than those in summer. Further research has been conducted for the heavy metals in sediment, pore water and shallow groundwater to explore the source of transport for the heavy metals. The relationship of single heavy metal concentrations between stream water pore water and shallow groundwater has been analyzed; there is a good positive agreement among these concentrations (Table 8). These demonstrate that the concentrations of heavy metals have homology, and the existence of heavy metal transportation between the interactions of these interfaces. The distribution of heavy metals in fluid-solid phase is determined by the driving force between precipitate and a complexing agent [49]. Organic complexing agents in water and sediments negate the dilution effect of heavy metals, which can increase the pore water, river water, shallow groundwater dissolved heavy metals; and precipitants tend to cause some partially dissolved heavy metals in water to transfer to sediments with the solid phase. The heavy metals in the hyporheic zone are not only influenced by the physiochemical conditions [50], but also closely related to the chemical features of stream water, pore water and surrounding groundwater (Table 7). There are apparent seasonal differences of heavy metals in overlay water, and the tendency in spring is significantly higher than in summer (Table 7). The relationship between heavy metals in stream water and pore water yields a significant positive agreement. Heavy metals have apparent seasonal variations, however, significant changes of the grain order size profiles are not shown (Figure 2). 

Therefore, the seasonal change of heavy metal concentrations in the sediments was mainly determined by the discrepancies between heavy metal concentrations in stream water. However, in certain experimental cases, the existence of heavy metals has greater discrepancies in the fluid phase and sediments, which may be related to the conditional distributions of stream bed sediments that differ from lake bed, the fluvial system easily influenced by flood and storm events, so the allocation, stability and balance of the heavy metals in fluid-solid phase should display a time scale [51]. River sediments in different climatic conditions, the occurrence of floods, intensity and frequency have larger discrepancies, the scoring and carrying capacity are different, the sediments’ structure and disturbing depths are also influenced by human activities such as flood discharges [52]. The precipitation and storm events are mainly concentrated from June to October; this leads to frequent erosional and semimetal conditions and degrees. The streambed sediments are relatively stable in spring and winter. All this may be the reason leading to the seasonal variations of heavy metals. 

The vertical variations of heavy metals have significant differences with the depths [53]. The sediment and grain size analysis show that the grain size order profiles differ with the depth. There is a significant stratification of the sediment in vertical depth (Figure 2d–f). The grain size of the sediments mainly correspond to clay and silt. Previous studies have reported that the heavy metal concentrations and chemical characteristics in the streambed sediments are influenced by the regional rainfall intensity, runoff erosion and carrying capacity. On the other hand, the concentration of heavy metals in the sediment is associated with hydraulic and exchange conditions and degree of contamination in the stream sediment. The single heavy metals were significantly associated with vertical variations of depth. This is evidenced by related study [38] that there was significant exchange with vertical sediments in the hyporheic zone. The hyporheic exchange is one of great important dynamic effects leading to a significant agreement between single heavy metals and vertical sediments.

Correlation analysis was used in comparisons of concentration of heavy metals combined with TP, TN, OM and grain size, and it was found that the concentration of heavy metals has moderate correlations with OM and grain size, however, there is specific and indirect relationship between heavy metals and TP and TN (Table 9). The grain size of streambed sediments is not the main controlling factor that determines the heavy metal concentrations. The heavy metals Zn and Cd have moderate agreement with OM, but the OM is not the controlling factor of these two heavy metals.

**Table 9:** The correlation matrix between heavy metals and OM, TP, TN, silt and clay in streambed sediments.

Item	Cu	Zn	Pb	Cd
Zn	0.21 **			
Pb	0.42 **	0.07		
Cd	0.12	0.27 **	0.24 **	
OM	–0.03	0.49 **	0.01	0.33 **
TP	–0.16	–0.03	0.10	0.08
TN	0.13	–0.06	0.14	–0.10
Silt and Clay	–0.15 *	0.18 **	–0.20 **	0.14 *

*N* = 219, * *p* < 0.05, ** *p* < 0.01 (Two-sided test).

### 3.3. The Evaluation of the Heavy Metals Environmental Risks in the Sediments

#### 3.3.1. Geo-accumulation Index Evaluation Results 

Due to the benthos activities ranging from 0 to 30 cm, it is very necessary to use the geo-accumulation index (I_geo_) to evaluate the accumulation of the heavy metals in different depths of the sediments in the study area in different seasons. The evaluation results displayed that I_geo_ values were greater in spring and winter than in summer in all study spots except the Huaxian gauge. It also found that the I_geo_ values of all the heavy metals were less than 0 in summer, except the average I_geo_ values of Cd that were higher than 4 in different depths in the study area, and this result revealed that Cd was the main element contributing to the severe pollution level of the study area. Apart from the severe Cd contamination, the pollution levels of the heavy metals were obviously different with different river sections, and their I_geo_ and contamination levels were also different with depths, even in the same study spot. These results indicated that the pollution status of the heavy metals has significant spatial and seasonal characteristics in the sediments of the Weihe River.

#### 3.3.2. Potential Hazard Index Evaluation Results 

The potential ecological hazard index was used to evaluate the hazard levels of single heavy metals and the comprehensive hazard level of multiple heavy metals, including Cu, Zn, Pb and Cd. The evaluation results showed that, with reference to the coefficients of the potential ecological risk of a heavy metal ($E_{r}^{i})$, Cu levels were greater in spring and winter than in summer with the values being less than 30 in these two seasons, indicating a light hazard level in the study area, except at the Huaxian gauge, where that the coefficient of the Cu were bigger than 30 in some depths in summer, indicating a moderate hazard level. As for Zn, the risk coefficients were less than 30 in the study area, indicating light pollution, though the coefficients were larger in spring and winter than summer. The risk coefficients of Pb were similar to those of Cu, where the average values of the coefficients corresponded to the light hazard level with the coefficients in spring and winter being greater than in summer in the study area, except that the coefficients ranged from 30 to 60 in the 0–10 cm and 10–20 cm layers at the Huaxian gauge in spring, indicating a moderate hazard level. The risk coefficients of Cd reached to the serious hazard level in a few depths in the study area, and the rest were up to the most serious hazard level. From a perspective of the average vales of the risk coefficients, there were also clear seasonal differences, where the coefficients in spring and winter were greater than in summer at different depths in the study area, and these results were consistent with the variations of Cu, Zn and Pb. These results suggested that we should pay the most attention to the ecological hazard of Cd due to its extremely serious hazard level as a single heavy metal in the Weihe River. However, the results also confirmed that we should not ignore the hazards of Cu and Pb in spring and winter due to their moderate hazard level in some individual spots. The comprehensive index of potential ecological risk of multiple heavy metals (E_RI_) displayed that the ecological hazard reached very serious levels in different depths of the study area, and this was mainly because of the extremely serious ecological hazard of the Cd, which reached an amount of 96.4%–99.5% in the E_RI_.

#### 3.3.3. Quality Standard Index Assessment Results 

**Figure 3:**
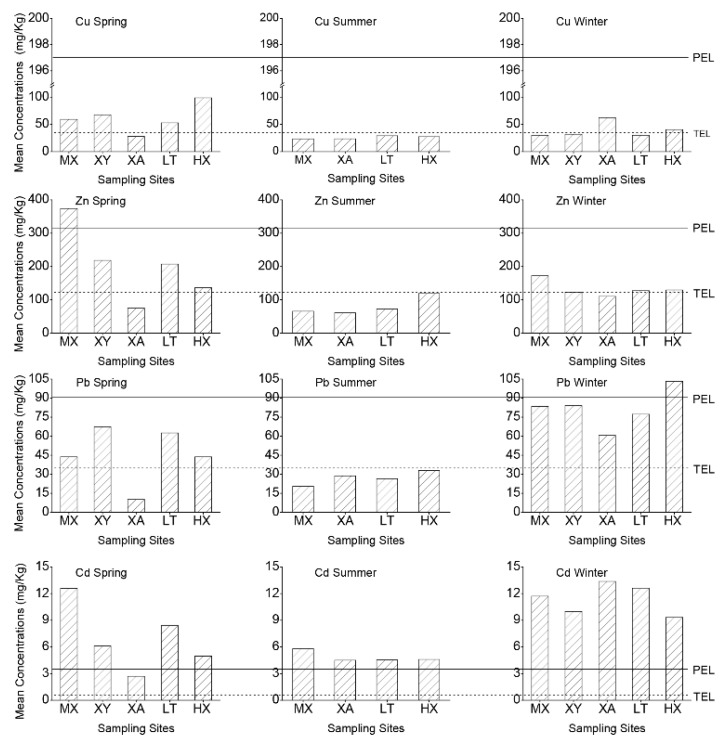
The effect threshold and mean concentrations of the heavy metals in streambed sediments in the Weihe River of Shaanxi Province (Vertical volume indicates the mean concentration of metals in sampling sites. MX: Meixian; XY: Xianyang; XA: Xi’an; LY: Lintong; HX: Huaaxian).

Considering the vertical character of benthos activities [54], this study took the mean maximum concentration of a heavy metal in a layer as evaluation value for pollution assessment (Figure 3). In the study temporal scale, the measurements of Cd concentrations are all greater than the PEL value, based on the evaluated categorical standard, indicating a strongly polluted degree. Generally, in spring, the measued concentrations of the rest of the heavy metals are concentrated in the range between TEL and PEL, corresponding to a moderately polluted degree. In summer, the concentrations of the heavy metals are the smallest among the seasons, and the heavy metals measurements indicate a lightly polluted degree. In winter, the vast majority of the measurements of the Cu and Zn are less than the EPL, and the heavy metals concentration is below the light degree. For the concentration of Pb, the measurement values are mainly distributed in the range of EPL and TPL, indicating a moderate pollution degree.

## 4. Conclusions

Chironomids and flutter earthworm were the dominant benthic species of the Weihe River in Shaanxi Province, whose activity areas mainly ranged from 0 to 20 cm with maximum depth up to 30 cm. All in all, the benthic biomass decreased with depth.

The concentrations of heavy metals in surface water and pore water have substantial seasonal differences in spring, summer, and winter in the Weihe River. In general, heavy metal concentrations were significantly higher in spring and winter than that in summer. Contrasting the concentrations between winter and spring, the metal concentrations show significant discrimination. The concentrations of heavy metals have vertical variations with the depths. 

Overall, the evaluation results from the three methods showed that the pollution levels of Cu, Zn, Pb and Cd were more severe in spring and winter than in summer, and the heavy metal pollutions varied with depths. The concentrations of Cu and Cd were greatly higher than the CMC, which would produce great toxicity effects to benthos. The toxicity effects of Pb and Zn displayed seasonal and spatial differences, and showed a positive correlation between the single heavy metal in surface water and pore water. The pollution level of the Cd was the most serious of the four heavy metals. However, for Cu, Zn and Pb, the results of the three methods were different, which was caused by the different focuses of these three methods.

We should undertake further research on the heavy metal response mechanism for the hydrological characteristics in the hypotheic zone, and clarify the migration and conversion mechanism of heavy metals in the solid-liquid two-phase system in the river. The dynamical monitoring and assessment of the heavy metals should be improved to provide the reliable data for heavy metal source tracing, heavy metal pollution prevention and polluted site restoration, and protecting the ecological balance and establishing the three-dimensional evaluation system for heavy metals in the stream.
