# Correction: Song, J.; Yang, X.; Zhang, J.; Long, Y.; Zhang, Y.; Zhang, T. Assessing the Variability of Heavy Metal Concentrations in Liquid-Solid Two-Phase and Related Environmental Risks in the Weihe River of Shaanxi Province, China. *Int. J. Environ. Res. Public Health* 2015, *12*, 8243-8262.

**DOI:** 10.3390/ijerph121114327

**Published:** 2015-11-12

**Authors:** Jinxi Song, Xiaogang Yang, Junlong Zhang, Yongqing Long, Yan Zhang, Taifan Zhang

**Affiliations:** 1 State Key Laboratory of Soil Erosion and Dryland Farming on the Loess Plateau, Institute of Soil and Water Conservation, Chinese Academy of Sciences, Yangling 712100, China; 2 College of Urban and Environmental Sciences, Northwest University, Xi’an 710027, China; E-Mails: yxgyff166@163.com (X.Y.); junlongzhangcq@hotmail.com (J.Z.); sjzxlyq@nwu.edu.cn (Y.L.); zhangy.11b@igsnrr.ac.cn (Y.Z.); zhangtaifan@163.com (T.Z.)

The authors wish to make the following corrections to this paper [1].

In the Acknowledgments Section, the sentence “This work was support by National Natural Science Foundation of China (Grant No.51379715)” should read as follows: 
